# Supplementary material for: Risk Stratification for Pacemaker Implantation after Transcatheter Aortic Valve Implantation in Patients with Right Bundle Branch Block
Source: J Clin Med. 2022 Sep 22;11(19):5580. doi: 10.3390/jcm11195580 (PMC9571112; doi:10.3390/jcm11195580)
Supplement: Supplementary file 1 [file jcm-11-05580-s001.zip › jcm-1902627-supplementary.pdf]

**Supplemental Table S1.** **PPI indications of patients with pre-existing RBBB before TAVI**
Third degree AV block
Second degree type 2 AV block
Alternating bundle branch blocks
Symptomatic bradycardia

Supplemental Table S2. **Baseline ECG characteristics of patients (RBBB, no PPI, survivors and non-survivors)**

	**All Patients (n=98)**	**Survivors (n=83)**	**Non-Survivors (n=15)**	**P Value**
Sinusrhythm	72 (73.5)	63 (75.9)	9 (60)	0.199
Heart rate	71 ±10	72 ±13	75 ±5	0.530
PQ duration (ms)	189 ±48	188 ±48	194 ±53	0.357
PQ ≥240ms	8 (11.1)	6 (7.2)	2 (13.3)	0.257
QRS duration (ms)	141 ±15	140 ±14.9	143 ±14	0.248
QRS ≥140ms	43 (43.9)	34 (41)	9 (60)	0.172
QT duration (ms)	424 ±36	423 ±38	429 ±29	0.511
QTc duration (ms)	465 ±27	462 ±25	480 ±29	0.100
PQ≥240ms + QRS≥140ms	6 (6.1)	5 (6)	1(6.7)	0.924
RBBB morphology				0.275
rsR’	68 (69.4)	57 (68.7)	11 (73.3)	
RsR’	8 (8.2)	5 (6)	3 (20)	
rSR’	18 (18.4)	17 (20.5)	1 (6.7)	
Rsr’	2 (2.0)	2 (2.4)	-	
Rsr’	2 (2.0)	2 (2.4)	-	
r-s-duration (ms)	46 ±15	49 ±14	50 ±19	0.517
s-r’-duration (ms)	98 ±15	98 ±15	98 ±11	0.753

Values are mean ± or n (%)

PPI=permanent pacemaker implantation

Supplemental Table S3. Degree of calcification in mm³ in CTA

	All Patients (n=53)	PPI after TAVI (n=20)	No PPI after TAVI (n=33)	P Value
Calcification AV region				
Non-coronary cusp	475 ±335	418 ±338	509 ±333	0.147
Right coronary cusp	357 ±256	318 ±182	379 ±241	0.267
Left coronary cusp	296 ±220	252 ±201	323 ±230	0.115
Calcification entire LVOT				
Non-coronary cusp	141 ±154	94 ±141	52 ±161	0.036
Right coronary cusp	9 ±9	3 ±9	4 ±10	0.312
Left coronary cusp	118 ±86	55 ±118	34 ±59	0.422
Calcification upper LVOT				
Non-coronary cusp	15 ±45	15 ±28	16 ±53	0.207
Right coronary cusp	2 ±8	2 ±6	3 ±8	0.695
Left coronary cusp	8 ±14	8 ±12	8 ±15	0.228

Values are mean ± or n (%)

PPI=permanent pacemaker implantation, CTA=computed tomography angiography

Supplemental Table S4. **Degree of calcification in CTA, subcategorized after tertiles**

	All Patients (n=53)	PPI after TAVI (n=20)	No PPI after TAVI (n=33)	P Value
Calcification AV region				
Non-coronary cusp				0.074
1st tertile	17	10	7	
2nd tertile	18	4	14	
3rd tertile	18	6	12	
Right coronary cusp				0.497
1st tertile	17	8	9	
2nd tertile	18	7	11	
3rd tertile	18	5	13	
Left coronary cusp				0.094
1st tertile	17	10	7	
2nd tertile	18	5	13	
3rd tertile	18	5	13	
Calcification entire LVOT				
Non-coronary cusp				0.022
1st tertile	17	2	15	
2nd tertile	18	8	10	
3rd tertile	18	10	8	
Right coronary cusp				0.376
1st tertile	17	6	11	
2nd tertile	18	9	9	
3rd tertile	18	5	13	
Left coronary cusp				0.376
1st tertile	17	6	11	
2nd tertile	18	5	13	
3rd tertile	18	9	9	
Calcification upper LVOT				
Non-coronary cusp				0.652
1st tertile	17	5	12	
2nd tertile	18	7	11	
3rd tertile	18	8	10	
Right coronary cusp				0.765
1st tertile	17	6	11	
2nd tertile	18	8	10	
3rd tertile	18	6	12	
Left coronary cusp				0.341
1st tertile	17	4	13	
2nd tertile	18	8	10	
3rd tertile	18	8	10	

Values are mean ± or n (%)

PPI=permanent pacemaker implantation, CTA=computed tomography angiography

Supplemental Table S5. **Annulus diameter among different groups**

	All Patients (n=98)	PPI after TAVI (n=43)	No PPI after TAVI (n=55)	P Value
Annulus size (mm)	24 ±2.0	23.5 ±2.0	24.4 ±1.9	0.059
	**All Patients (n=98)**	**Male (n=42)**	**Female (n=56)**	**P Value**
Annulus size (mm)	24 ±2.0	25.1 ±2.0	22.3 ±1.4	<0.001

Values are mean ± or n (%)

PPI=permanent pacemaker implantation, CTA=computed tomography angiography

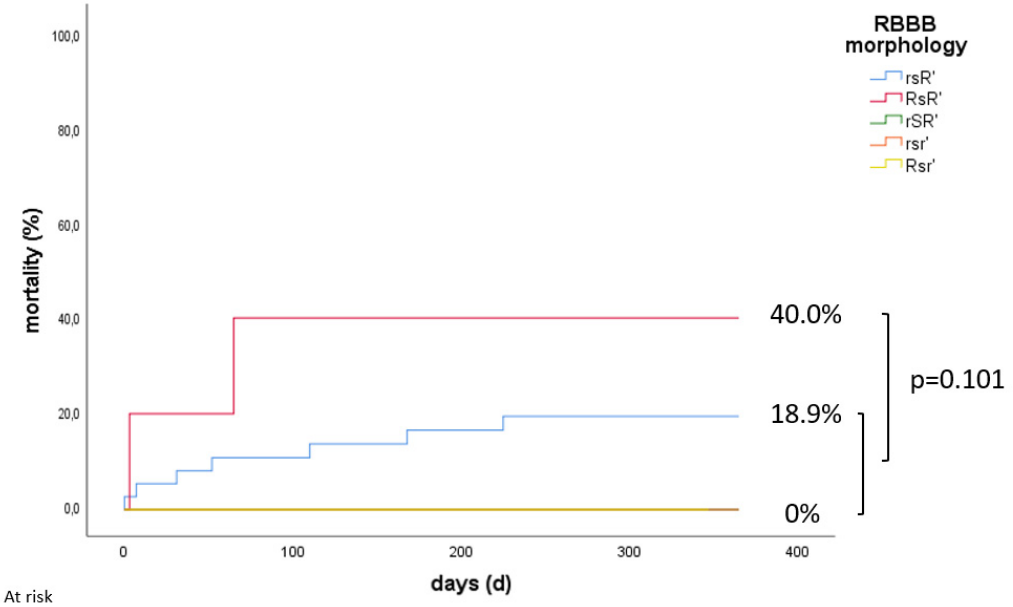

No. At risk				
rsR‘	37	31	29	28
RsR‘	5	3	3	3
rSR‘	10	10	10	1
rsr‘	2	2	2	2
Rsr‘	1	1	1	1

Supplementary Figure S1. 1-year mortality in patients with RBBB and no PPI after TAVI in relation to RBBB morphology (p=0.286)

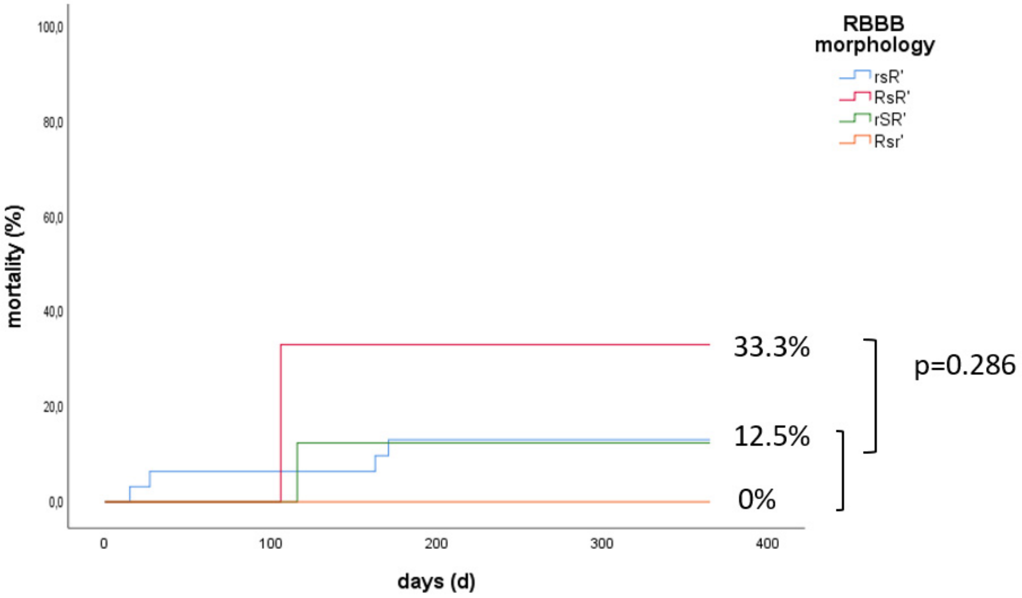

No. At risk				
rsR'	31	29	26	26
RsR'	3	3	2	2
rSR'	8	8	7	7
rsr'	0	0	0	0
Rsr'	1	1	1	1

Supplementary Figure S2. 1-year mortality in patients with RBBB and PPI after TAVI in relation to RBBB morphology (p=0.738)
